# New Appliances and Things Medical

**Published:** 1894-03-31

**Authors:** 


### NEW APPLIANCES AND THINGS MEDICAL.

[All preparations, appliances, novelties, &c., of which a notice is desired, should be sent for the Editor, to care of The Manager, 428, Strand, London, W.C.]

### COCA TONIC CHAMPAGNE (LAURENT-PERRIER, GRAND VIN BRUT). "CHAMPAGNE SANS SUCRE, LAURENT-PERRIER."

(Consignees, Hertz and Collingwood, 4, Sussex Place, Leadenhall Street, E.C.

Although the extract and other preparations of coca have long been known to the public in this as well as in other countries, it was not until the meeting of the British Medical Association at Newcastle, that a preparation of this drug blended with palatable alcoholic beverage was offered to the profession. The method of manufacture as carried out by the well-known firm of Laurent-Perrier et Cie. is as follows. The juice of the grape is run into casks, and fermentation allowed to take place, a process which is followed by the precipitation of the heaviest sediment. The supernatant fluid is racked into casks, and allowed to remain for a few months, during which time the fermentation slowly continues. The various samples are then blended, and the following spring the process of fermentation recontinued. After this process is completed Erythroxylon Coca extract is added, together with a small quantity of syrup, and then bottled. Thus it is left for a couple of years. Then cleared of all sediment and rebottled. The finished wine is a champagne of good quality (about 12 per cent. alcohol), pleasant flavour, with a slight taste of coca. Our analysis shows the merest trace of unconverted sugar, and hence the wine may be safely prescribed in cases of gout, diabetes, &c., when sugar-containing wines are usually forbidden. Coca being thus agreeably blended with an alcoholic beverage, an immediate stimulant is happily combined with a more lasting tonic effect. In cases of neurasthenia, neuralgia and allied conditions it is very serviceable, as well as in other cases where a tonic and stimulant effect is desirable. The same firm supply the champagne without the coca extract, and being a wine free from sugar, it is especially adapted for gouty and diabetic invalids.

### MICROBENE.

(Weston, Hunt, and Co., 39, Seething Lane, E.C.)

This is an antiseptic which the manufacturers hope will largely replace carbolic acid for general purposes. In the hands of experts or public bodies that is hardly likely to be the case. For domestic use, however, it is probably quite as efficacious, and has the additional merit of being non-corrosive. Moreover, in reasonable quantities it is calculated to do little harm should it be swallowed by mistake or intentionally, and hence is a far safer antiseptic agent than carbolic acid. The fluid itself contains something like 30 per cent. of tar acids, it is a dark, oily-looking fluid, which mixes readily with water, forming an emulsion or milky solution, not altogether unlike that formed when liquor carbonis detergens is poured into water. It is a good deodoriser, and especially useful for cleaning sinks, traps, &c. It is recommended for veterinary use, and its tarry nature renders it efficacious in the treatment of mange, eczema, and other skin diseases. Insects, lice, maggots, &c., appear to avoid places which have been scrubbed down with a solution of Microbene. The "coal tar soap" manufactured by the same firm is formed of a basis of ordinary domestic soap, into which 10 per cent. of Microbene has been intimately mixed by the "cold process." It is a soap which offers many advantages for scrubbing boards, &c., in dormitories of workhouses and similar institutions, where more expensive and vigorous antiseptic measures cannot be adopted. "The anti-prickly heat soap" is a specially prepared soap, to which Microbene has been added. It is by no means a disagreeable soap for personal use, but whether it has any direct influence on the prevention of urticaria beyond keeping the ducts of sweat and sebaceous glands in a healthy condition, it is difficult to say. Urticaria may arise from such diverse exciting causes that it is often difficult to predict what may prove efficacious in its treatment. We are, therefore, by no means inclined to dissuade sufferers from giving this soap a trial.

